# Rehabilitation of an Advanced Case of Adenoid Cystic Carcinoma

**DOI:** 10.1155/2015/651321

**Published:** 2015-01-29

**Authors:** Luiz Evaristo Ricci Volpato, Lorena Frange Caldas, Paulo Henrique de Souza Castro, Artur Aburad de Carvalhosa, Maria Carmen Palma Faria Volpato, Matheus Coelho Bandéca, Álvaro Henrique Borges

**Affiliations:** ^1^University of Cuiabá, Avenida Manoel José de Arruda 3.100, 78065-900 Cuiabá, MT, Brazil; ^2^Mato Grosso Cancer Hospital, Avenida Historiador Rubens de Mendonça, 78.055-500 Cuiabá, MT, Brazil

## Abstract

Adenoid cystic carcinoma is a cancer of the salivary gland that primarily affects the parotid, submandibular, and accessory salivary glands. Its growth is slow and it has infiltrative nature. A 46-year-old female patient coming from the rural area presented a lesion on the palate and reported pain in the region for three years. After incisional biopsy, and histopathological diagnosis of adenoid cystic carcinoma of the cribriform type of minor salivary gland, superior hemimaxillectomy and adjuvant treatment with radiotherapy and maxillofacial prosthetic rehabilitation were performed.

## 1. Introduction

Adenoid cystic carcinoma (ACC) is a salivary gland tumor that may originate in either major or minor salivary glands. ACC has a preferential location in the hard/soft palate, predominantly in females [1, 2].

In the clinical assessment it is shown as a nodule of hardened consistency, fixed on palpation and covered with intact mucosa [2–4]. The ACC is a neoplasm that expresses two neoplastic cell components: ductal epithelial and myoepithelial cells; mimicking the intermediate duct of the salivary glands, its parenchyma may show tubular, cribriform, or solid morphologic pattern [5].

It is a slow growing and painless mass; however, pain symptoms may be present due to the predisposition to invade tissues and nerves [4, 5]. The infiltrative characteristic of the ACC is responsible for local and contiguous spread and reoccurrence that can happen years after the initial radiotherapy and surgical treatment [5].

Treatment for ACC consists of four distinct modalities: surgical therapy, radiotherapy, chemotherapy, and combined therapies (surgery and radiotherapy; radio and chemotherapy). The latter has been the modality of choice in most cases, because only surgical removal or radiation therapy does not discard the possibility of recurrence in the margins nor metastases to occur mainly in the cervical lymph nodes, lungs, bones, and brain [5–7].

Oncologic surgical procedures to remove the tumor, associated with complementary therapeutic procedures, generate temporary or permanent mutilation in the face of patients causing physiognomy deformations and functional changes [6].

If surgical reconstruction is not possible, prosthetic rehabilitation is indicated aiming to correct the difficulties of feeding, swallowing, and speech, improve the aesthetic appearance, and conduct social and professional reintegration of the patient, enhancing quality of life [8, 9].

This paper presents the case of a patient with adenoid cystic carcinoma since her (late) diagnosis to her rehabilitation.

## 2. Case Report

**Figure 1:**
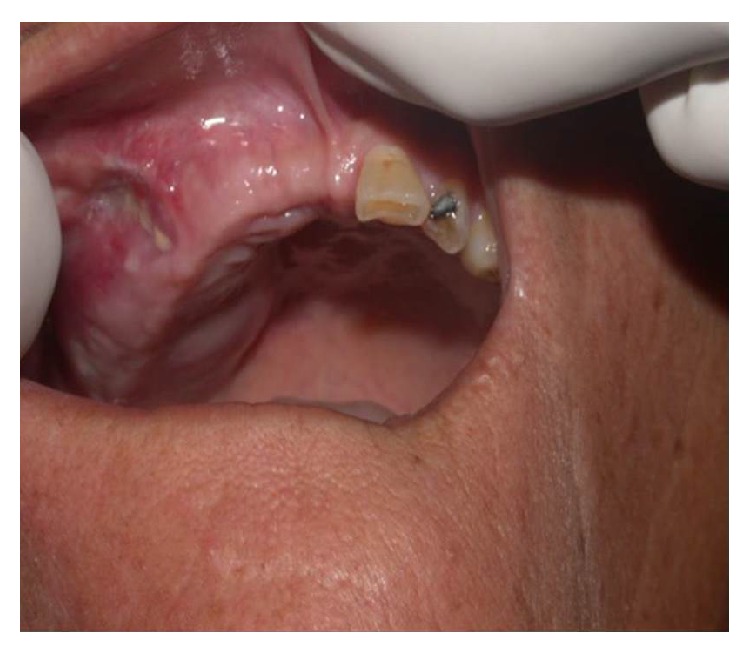
Initial aspect of the injury.

A 46-year-old female patient, from the rural area of the state of Mato Grosso, Brazil, was examined in an oral cancer prevention campaign and presented a lesion in the right side of the hard palate (Figure 1). She reported pain in the region with three years of evolution.

The main complaint was pain for three years and volumetric enlargement on the right side of the maxilla. During anamnesis, the patient reported that when the pain started, it was intense and localized, ceasing with the use of analgesics. Faced with this complaint, she sought a dentist in her municipality. As there was no availability of imaging, the professional's conduct was extraction of the upper right side posterior teeth of the patient. Such procedure contributed to the worsening of pain and began the expansion of the maxillary alveolar ridge. Subsequently, the possibility was considered that it was a trigeminal neuralgia and the patient was referred for investigation. However, before consulting with the neurologist, she was examined in the oral cancer prevention campaign where, after collecting the clinical history, an incisional biopsy of the lesion was performed and the material was sent for pathological examination.

**Figure 2:**
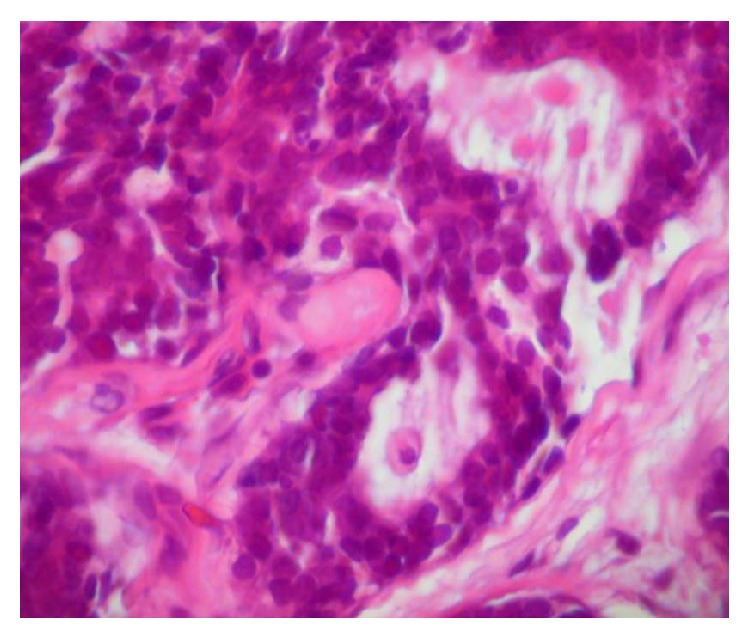
Photomicrograph showing the cribriform pattern of the ACC.

Histological sections revealed ectodermal tumor fragment originating from the salivary gland formed by cribriform parenchyma, forming structures resembling ducts consisting of two cell layers, the innermost layer interpreted as ducal neoplastic cell and the outermost layer is interpreted as being composed of neoplastic myoepithelial cells (Figure 2). Stroma consisting of loose fibrous connective tissue completed the histology leading to the final diagnosis of adenoid cystic carcinoma cribriform type of minor salivary gland.

**Figure 3:**
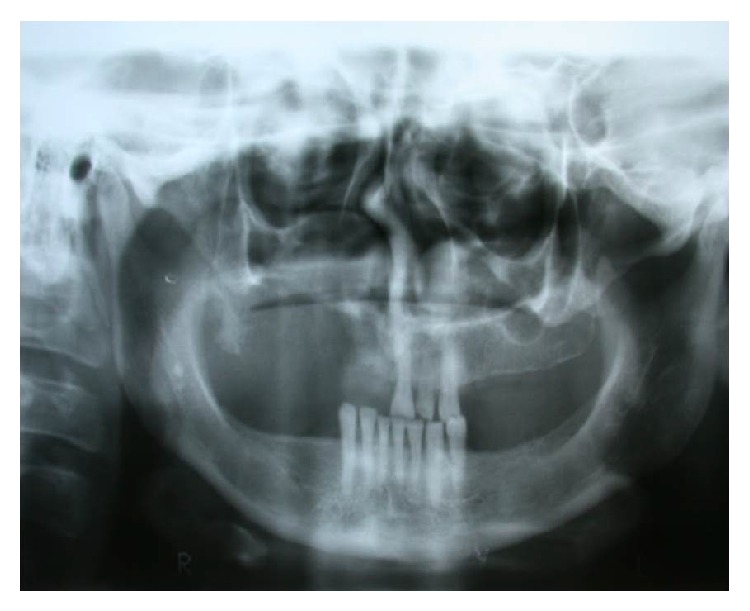
Panoramic radiography.

**Figure 4:**
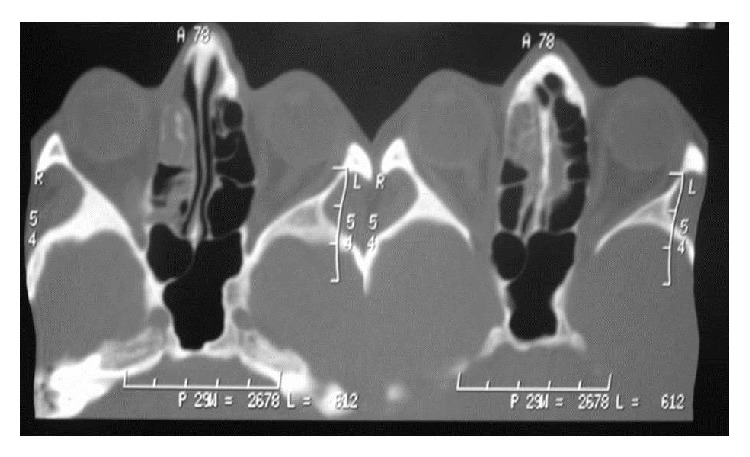
CT section of the region.

**Figure 5:**
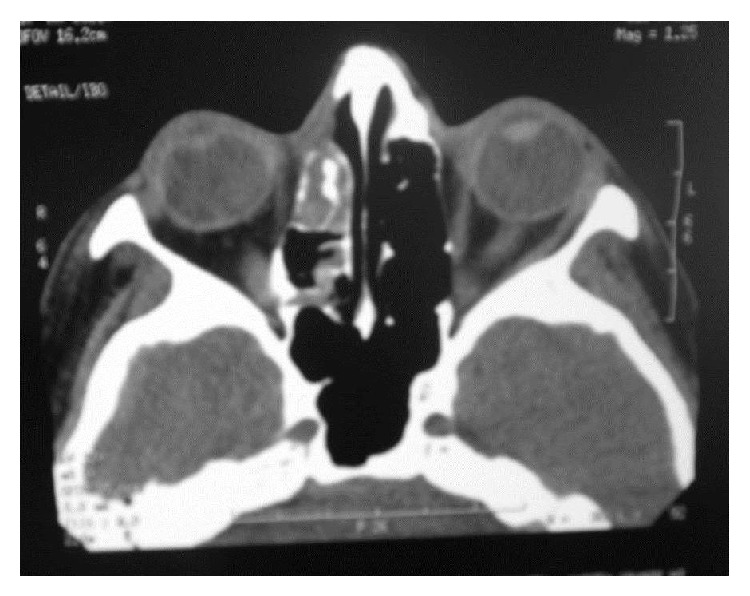
CT section of the region.

The patient was then referred for treatment in Cuiabá, the state capital, where additional tests were conducted. The panoramic radiograph (Figure 3) and computed tomography (CT) (Figures 4 and 5) revealed invasion of the maxillary bone, maxillary sinus, ethmoid bone, nasal cavity, frontal sinus, and central nervous system, frontal lobe of the cerebrum. CT of the sinuses revealed lesion in the upper part of the mouth on the right side with involvement of the gingiva, maxillary sinus, and right nasal fossa with posterior extension to the pharynx, involving the trigone retromaxillary, pterygoid muscle, and pterygopalatine fossa. Magnetic resonance imaging of the face showed anomalous tissue compromising the right side of the face, suggesting expansive and infiltrative lesion predominantly solid with signs of small cystic component in the superior and medial portion of indeterminate lineage. Video-laryngoscopy showed compatibility with tumor on the hard palate. Chest radiography showed up normal. Final TNM classification was T4N0M0.

**Figure 6:**
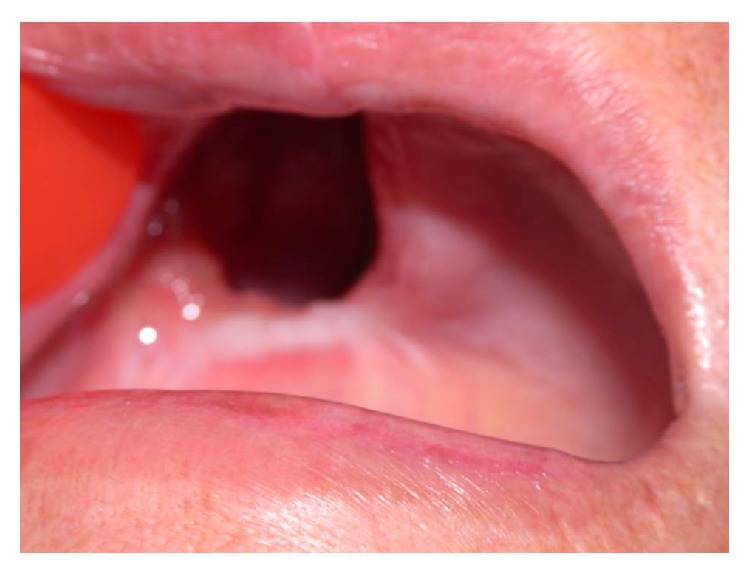
Clinical aspect after treatment.

**Figure 7:**
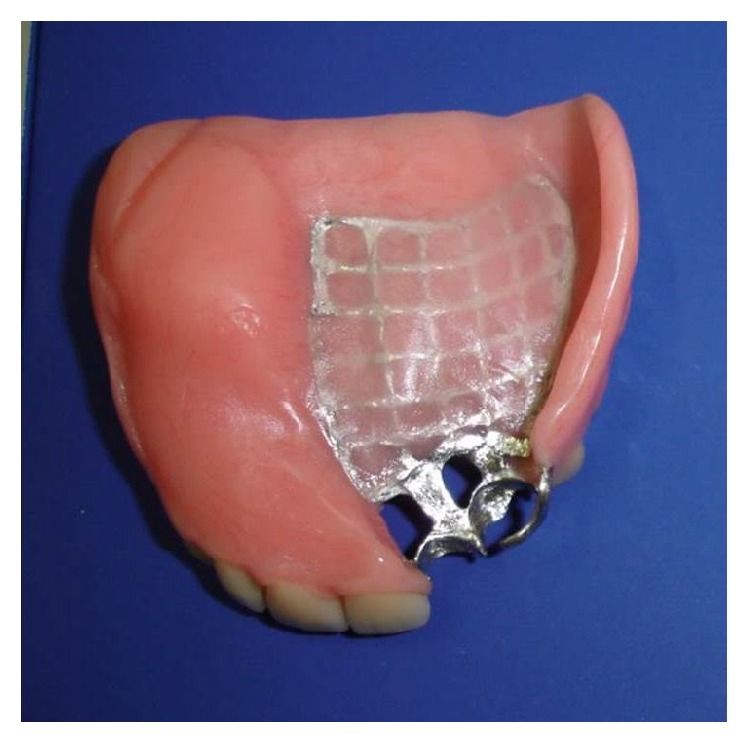
Obturator prosthesis.

The patient was subjected to surgical treatment with hemimaxillectomy of the affected side and underwent postoperative radiotherapy. Reconstructive surgery was performed nine months later (Figure 6). As surgery was not able to close the communication between oral and nasal cavities, the patient was rehabilitated with an obturator prosthesis (Figure 7).

After monitoring for four years, the patient died.

## 3. Discussion

Malignant salivary gland neoplasms are relatively uncommon, accounting for less than 7% of cancers of the head and neck. Of these, only 10% are diagnosed as ACC [3, 4]. This neoplasm presents numerous clinical and microscopic characteristics similar to other tumors; thus differential diagnosis should always be carried out by histological and immunohistochemical exams [7, 8]. The differences between the ACC and other glandular tumors, such as adenocarcinoma, are only revealed by specific markers of collagen IV, laminins and integrins. These characteristics demonstrate that the diagnosis must be made quickly in order to establish the most appropriate treatment [7, 10].

Microscopically, the ACC can present itself in three distinct forms: tubular, cribriform, and solid, or even the same tumor can present the three microscopic patterns; there is, however, the predominance of one of them. There is a clear correlation between the aggressiveness of the lesion, the microscopic type, and the stage of tumor development, with the prevalence of poor prognosis for solid tumors followed by cribriform and tubular [8–10]. This is corroborated by the presented case, an aggressive tumor consisting of sometimes solid (with tendency to form ducts or cystic spaces) and sometimes cribriform parenchyma formed by two cell types: ductal epithelial cells and neoplastic myoepithelial.

Other factors that can also influence the prognosis of ACC are the size and location of the tumor, the type of therapy used, the dose of radiation, and the presence of metastasis [11, 12]. The case presented had a late diagnosis reflecting an infiltrative lesion that compromised the right face in the medial and inferior portion of the maxillary sinus ascending to the medial margin of the orbit. Given this clinical picture, radiotherapy and hemimaxillectomy were performed.

The ACC has a very infiltrative and poorly exophytic growth; that is, it invades more and in usual inspection it looks like a small nodule, suggesting fibroma, or as a benign lesion that most often no mucosal ulceration is observed. This feature, added to its low incidence, can often lead to misinterpretations or delay in diagnosis [10]. The lack of resources, like imaging, also contributes to mistakes and delay in diagnosis. The discreet but very invasive lesion of this case with at least three years of evolution in a patient residing in the rural area of a small municipality with little access to specialized professionals and imaging reflect the difficulty in establishing an early diagnosis and prompt treatment for ACC.

The evaluation of salivary gland tumors has been performed by various imaging modalities, but multidetector computed tomography and magnetic resonance imaging are particularly useful in determining the extent of the tumor and the possibility of metastasis in lymph nodes. In addition, multidetector computed tomography with contrast allows the surgeon to determine whether the lesion is cystic or solid [3, 4]. When they reach the bony portion, ACC have spinal dissemination, thereby promoting no radiographic findings to substantiate their degree of commitment. Intracranial extensions of ACC range between 4 and 22% of cases. Depending on the original site of the tumor, three mechanisms are involved: direct extension by bone destruction, through the blood, and through the perineurium and endoneurium being the trigeminal nerve, the cranial nerve most commonly affected [11]. The median time of local recurrence is five years while distant recurrences oscillate around seven years, with the primary site of distant metastasis lung, followed by brain and bone [11].

The mutilation caused by delayed treatment of cancer in the region of the face can cause much inconvenience to the patient of aesthetic, functional, and emotional natures, impacting in the self-esteem and often leading to social isolation [12]. In the presented case, even after undergoing reconstructive surgery, the patient still had oroantral communication and sinking of the face on the right side. The prosthetic rehabilitation improved her speech, chewing, and aesthetics as well as isolating the mouth from the nasal cavity greatly improving her quality of life.

It is important that health professionals do not underestimate the reports of their patients and engage in their diagnosis or immediate referral; as for malignant neoplasms, early diagnosis is critical to obtaining a favorable prognosis. In the other hand, when facing a poor prognosis, every effort should be directed to improve patient's quality of life.

## 4. Conclusion

Adenoid cystic carcinoma is a malignant neoplasm of difficult diagnosis and has high associated morbidity and mortality. The treatment executed in this case allowed the patient to live her last years with quality. However, her survival would be greater if the therapy was instituted earlier.
